# The Implementation of Randomization Requires Corrected Analyses. Comment on “Comprehensive Nutritional and Dietary Intervention for Autism Spectrum Disorder—A Randomized, Controlled 12-Month Trial, *Nutrients* 2018, *10*, 369”

**DOI:** 10.3390/nu11051126

**Published:** 2019-05-21

**Authors:** Colby J. Vorland, Andrew W. Brown, Stephanie L. Dickinson, Andrew Gelman, David B. Allison

**Affiliations:** 1 Department of Applied Health Science, School of Public Health - Bloomington, Indiana University, Bloomington, IN 47405, USA; awb1@iu.edu; 2 Department of Epidemiology and Biostatistics, School of Public Health - Bloomington, Indiana University, Bloomington, IN 47405, USA; sd3@indiana.edu; 3 Department of Statistics, Columbia University, New York, NY 10027, USA; gelman@stat.columbia.edu

We commend Adams et al. [1] for undertaking a lengthy intervention with a randomized design. However, we have concerns about the implementation of the randomization and analysis of results.

Using block randomization, the authors enrolled 37 participants to the treatment and 30 to the non-treatment groups. In response to our email asking how block randomization led to these discrepant group sizes, the corresponding author added that they balanced participants between high and low “ABA therapy” and by age, and paired siblings to the same treatment groups, then randomly assigned within each batch. We thank the author for communicating these details; however, this raises three specific concerns regarding the randomization and/or the extent to which the analyses used are correct given the randomization.

First, the randomization actually used was not described in the published report. Specifically, the authors reported that participants “were enrolled on a rolling basis, and participants with similar ages were matched and then randomly assigned to one of the two groups” [1]. However, the authors clarified in a personal communication that randomization also involved balancing by level of ABA therapy, and randomly assigning within each batch, which were not reported.

Second, given that the authors also communicated to us that siblings were assigned the same treatment, this means that the independence of observations assumptions of ordinary least squares analyses (which include the analyses the authors used) is violated [2,3], and analyses must be adjusted to account for this clustering effect [4]. Observations within clusters are often correlated, and effects may be the result of other factors besides the treatment, such as the same family environment. As such, not accounting for clustering may cause understatement of uncertainty in the intervention effect, and analysis as a partially clustered design should be performed [5].

Third, although block randomization can be an appropriate design, block effects should be included in statistical analyses [6].

One additional concern was the curious duality that the authors utilized two-tailed t-tests for biomarker outcomes but one-tailed t-tests for behavioral outcomes based on a hypothesized directionality [1]. One-tailed tests are often inappropriate because treatments could potentially worsen outcomes [7], and the authors even describe examples of behaviors worsening [1]. Further, their previous study utilized two-tailed t-tests with several of the same behavioral measures [8]. Because this study was retrospectively registered [9], we cannot confirm whether the choice of test was an a priori analysis decision.

Because the data are not publicly available, we are unable to perform the corrected analyses. We therefore request that the authors do so, and that they amend their report to accurately and completely describe their methods in the spirit of rigorous and transparent research [10]. We offer our assistance conducting reanalysis if requested.
